# Hypoglycemic effects of dracorhodin and dragon blood crude extract from *Daemonorops draco*

**DOI:** 10.1186/s40529-024-00415-1

**Published:** 2024-03-06

**Authors:** Yung-Hao Ching, Fang-Mei Lin, Hong-Chi Chen, Ching-Yun Hsu, Sze Yen P’ng, Tai-No Lin, Yu-Chia Wang, Cheng-Jun Lin, Yi-Chi Chen, Tsung-Jung Ho, Hao-Ping Chen

**Affiliations:** 1 Department of Molecular Biology and Human Genetics, Tzu Chi University, 970374 Hualien, Taiwan; 2 Department of Biochemistry, School of Medicine, Tzu Chi University, 701, Sec 3, Zhongyang Road, 970374 Hualien, Taiwan; 3 Department of Biomedical Sciences and Engineering, Tzu Chi University, 970374 Hualien, Taiwan; 4 School of Post-Baccalaureate Chinese Medicine, Tzu Chi University, 970374 Hualien, Taiwan; 5 Department of Chinese Medicine, Tzu Chi University, 970374 Hualien, Taiwan; 6 Integration Center of Traditional Chinese and Modern Medicine, Hualien Tzu Chi Hospital, 970473 Hualien, Taiwan

**Keywords:** Blood glucose, Dracorhodin, Dragon blood, Jinchuang ointment, Wound healing

## Abstract

**Background:**

Dragon blood is a red fruit resin from the palm tree *Daemonorops draco* and is a herbal ingredient used in the traditional Chinese medicine, “Jinchuang Ointment,” which is used to treat non-healing diabetic wounds. According to the Taiwan Herbal Pharmacopeia, the dracorhodin content in dragon blood should exceed 1.0%.

**Results:**

Our findings indicate that dracorhodin and dragon blood crude extracts can stimulate glucose uptake in mouse muscle cells (C2C12) and primary rat aortic smooth muscle cells (RSMC). Dracorhodin is not the only active compound in dragon blood crude extracts from *D. draco*. Next, we orally administered crude dragon blood extracts to male B6 mice. The experimental group displayed a decreasing trend in fasting blood glucose levels from the second to tenth week. In summary, crude extracts of dragon blood from *D. draco* demonstrated in vivo hypoglycemic effects in B6 male mice.

**Conclusions:**

We provide a scientific basis “Jinchuang ointment” in treating non-healing wounds in patients with diabetes.

## Background

Over the past few decades, “Jinchuang ointment,” a traditional Chinese medicine (TCM), has demonstrated notable efficacy in managing chronic non-healing wounds in patients with diabetes in Taiwan (Ho et al. 2016). A porcine excisional wound model has been established to further substantiate its effectiveness (Ho et al. 2020). Additionally, a clinical study was conducted to assess the therapeutic potential of “Jinchuang ointment” in treating leprosy-associated chronic wounds (Hsu et al. 2019). This herbal medicines are formulated using ingredients such as catechu, dragon blood, frankincense, myrrh, borneol, camphor, starch, wax, and lard. Among these components, starch, wax, and lard are considered excipients.

**Fig. 1:**
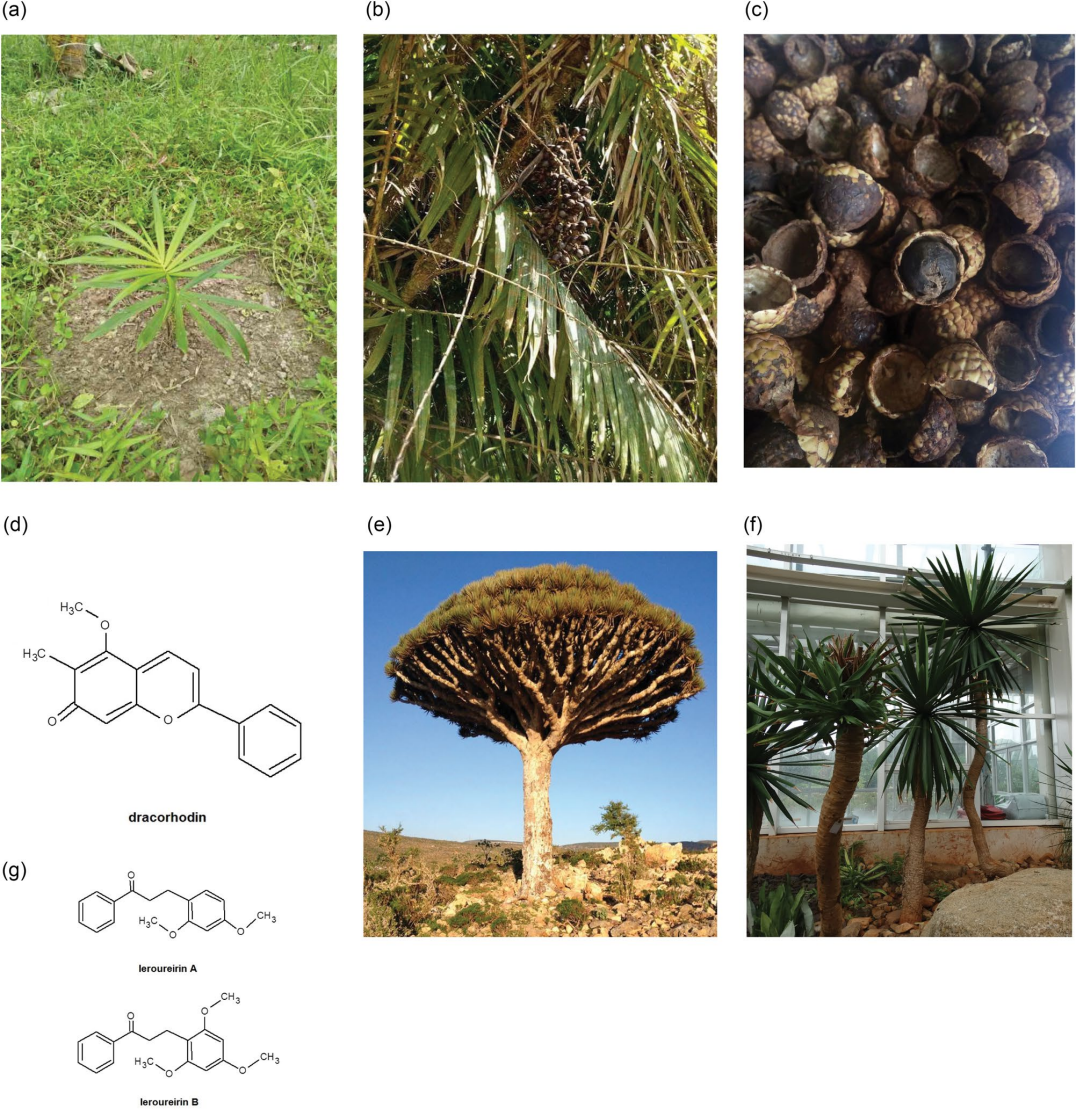
(**A**), (**B**) and (**C**) The *Daemonorops draco* palm tree on Sumatera Island, Indonesia. (**D**) Chemical structure of dracorhodin, the indicator compound in *D. draco*. (**E**) *Dracaena cinnabari*, on Socotra Island, Yemen. (**F**) *Dracaena cochinchinensis*, in Yunnan Province, China. (**G**) Chemical structure of leroureirins A and B, the indicator compounds in *D. cinnabari* and *D. cochinchinenesis*

Both dragon blood obtained from *Daemonorops draco* and catechu, derived from *Uncaria gambir* Roxb., exhibit potent in vivo and in vitro angiogenic activity, which is a critical aspect of wound healing (Krishnaraj et al. 2019; Ho et al. 2022). Dragon blood is derived from *D. draco* fruit, a tropical palm tree found in Southeast Asia (Fig. 1A and B) (Wu et al. 2019). According to the Pharmacopeia of the People’s Republic of China and Taiwan Herbal Pharmacopeia, the dracorhodin content in dragon blood should exceed 1% to be considered authentic. Dracorhodin is also an active ingredient responsible for angiogenesis (Fig. 1C). This prompts the question of why “Jinchuang ointment” is particularly effective in treating localized non-healing wounds in patients with diabetes. Insulin resistance and insufficient production are common causes of diabetes (Eckel et al. 2005). Therefore, it is plausible to ponder whether there an ingredient within “Jinchuang ointment” that possesses the capability enhances cellular glucose uptake.

Two distinct categories of plants, namely the families Asparagaceae and Arecaceae, produce dragon blood for use in folk medicine (Table 1). Notably, *Dracaena cochinchinesis -*derived dragon blood exhibits anti-diabetic activity (Ruxue et al. 2002; Gu et al. 2009). This dragon blood appeared in the TCM market in 1972; however, that used in “Jinchuang ointment” sourced from *D. draco* was published as early as in 1732. As such, *D. cochinchinesis -*based dragon blood is not the type used in “Jinchuang ointment.”

**Table 1:** Dragon blood-producing plants used in traditional Chinese medicine

Plant family	Plant species	Origin	History used in TCM
Asparagaceae	*Dracaena cinnabari*	Socotra Island, Yemen	Import into China via silk road after Tang Dynasty between AD 651 and AD 798. Silk road was closed until the 15th Century. Import was suspended.
	*Dracaena cochinchinensis*	Yunnan Province, China	Discovered in China after AD 1972 for the demand of treating wounds after the Vietnam war.
Arecaceae	*Daemonorops draco*	Indonesia and Malaysia	Import into China via sea transportation after Ming Dynasty, between AD 1368 and AD 1644 until now.

Given the two distinct plant species, there is considerable interest in exploring whether dragon blood from *D. draco* exhibits anti-diabetic activity. We report here, for the first time, that both dracorhodin and crude extracts of dragon blood from *D. draco* not only enhanced glucose uptake in mouse muscle cells but also reduced blood sugar levels in animal experiments.

## Methods

### Materials

2-(7-Nitro-2,1,3-benzoxadiazol-4-yl)-D-glucosamine (2-NBDG; catalog number: N13195) was purchased from Invitrogen™ (Waltham, MA, USA). Molecular biology-grade DMSO was purchased from Sigma-Aldrich (St. Louis, MO, USA). *D. perchlorate* (batch number: X-059-181114) was purchased from Chengdu Herb Purify Co., Ltd. (Chengdu, China). Dragon blood (commercial name: Draconis Sanguis; batch number: 712,041), containing 1.3% dracorhodin, was obtained from Ledstream Technology SDN BHD (Pulau Pinang, Malaysia). Oillio quick thickener was obtained from Shun Hwa Pharmaceutical (Taipei City, Taiwan). The inhibitors, LY294002 (catalog number: S1105) and dorsomorphin (Compound C, catalog number: S7840), were purchased from Selleck Chemicals, LLC. (Houston, TX, USA).

### Dragon blood crude extracts preparation

Dragon blood blocks were ground into a fine powder. Powder (1 g) was dissolved in methanol (10 mL). After sonication for 20 min and soaking overnight at room temperature, methanol extracts were transferred to new vials. Undissolved particles were removed by centrifugation at 4500 ×g for 5 min. The supernatant was dried in 1.5 ml Eppendorf tubes using a Savant SpeedVac™ Vacuum Concentrator (Thermo Fisher Scientific Inc. Waltham, Massachusetts, USA). The obtained solids were crude extracts of dragon blood dissolved in DMSO for subsequent in vitro experiments.

### C2C12 cell lines, primary cultures, and rat aortic smooth muscle cells

Murine skeletal muscle cell lines (C2C12 myoblasts) were kindly provided by MD Yu-Jen Chiu (Division of Plastic and Reconstructive Surgery, Department of Surgery, Taipei Veterans General Hospital). The cells were cultured in Dulbecco’s modified Eagle’s medium-high glucose (DMEM-H) (Gibco Co., Ltd., Grand Island, NY, USA) containing 10% (v/v) cosmic calf serum (Fisher Scientific, Waltham, MA, USA) and 1% penicillin–streptomycin (P/S; Gibco) at 37 °C under 5% (v/v) CO_2_ atmosphere until cells were 70–80% confluent. C2C12s were differentiated in DMEM-H medium containing 2% (v/v) horse serum and 1% P/S for five days. The medium was changed daily. Fully differentiated myotubes were used for subsequent experiments.

Primary rat aortic smooth muscle cells (RASMC) were isolated from the aorta of Sprague–Dawley rats (Edward P. Feener 1995). Cells were cultivated in Dulbecco’s modified Eagle’s medium-low glucose (DMEM-L) supplemented with 10% (v/v) fetal bovine serum (FBS; Gibco) and 1% P/S at 37 °C under 5% (v/v) CO_2_ atmosphere. The passage numbers of the cell cultures used in this experiment were between 8 and 15.

### In vitro glucose uptake assay

C2C12 cells were seeded in a 6-well plate at a density of 2 × 10^5^ cells/well and cultured in 1 ml medium. After incubating the cells in a CO_2_ incubator at 37 °C overnight, the medium was replaced with differentiation medium containing 2% horse serum to induce cell differentiation. The medium was changed daily, and on the fifth day, the desired concentration of dracorhodin or crude dragon blood extract was added to the culture medium for a 24-h incubation.

After incubation, the medium was removed and the cells were washed twice with 2 mL phosphate-buffered saline (PBS). Then, 1 ml of glucose-free DMEM containing 50 µM 2-NBDG was added to the cells, and cultured for 2 h. The DMEM was then removed, and the cells were washed twice with PBS. The cells were detached using 0.25% trypsin-EDTA (150 µl), centrifuged at 2000 ×*g* for 5 min, and the supernatant was removed. The cells were washed twice with 500 µl of PBS at 2000 ×*g* for 5 min Finally, the cells were resuspended in 500 µl PBS, and the fluorescence intensity was measured using a flow cytometer. The settings of the fluorescence detector were as follows: excitation wavelength, 487 nm; emission wavelength, 542 nm (Zou C 2005).

### Laboratory animal usage

The Institutional Animal Care and Use Committee of Tzu Chi University approved the animal experiments (approval number: 111,011). Four-week-old male C57Bl/6JNarl (B6) mice were purchased from the National Laboratory Animal Center (Nangang, Taipei, Taiwan). Known for their naturally elevated blood glucose levels, B6 mice, an inbred strain, serve as a common animal model of type 2 diabetes. Before commencing the experiment, the mice were acclimated four-week acclimation period post-importation to prevent anxiety-induced blood glucose elevation. They were housed in an IVC facility maintained at 22 °C under a 12-h light and dark cycle, with *ad libitum* access to autoclaved water and standard rodent chow (Laboratory Autoclavable Rodent Diet 5010, Purina Mills Inc.).

The daily recommended oral dosage of dragon blood in a adult man is 33 mg/kg. To adjust for mice, we applied an allometric scaling method based on body surface area, yielding a dosage of 405.9 mg/kg (Nair 2016). Considering the average weight of 25 g for an 8-week-old adult B6 male, the daily dragon blood dose for each mouse was 31 mg.

The red resin of the dragon blood is water-insoluble; therefore, the dragon blood was ground into a fine powder. To administer the dragon blood suspension, we prepared a solution with a final concentration of 0.435 g/ml daily immediately before feeding. This may be because the compounds in the dragon blood are susceptible to oxidation. Subsequently, the finely ground powder was reconstituted in an Oillio thickening agent (Shun Hwa Pharmaceutical Co., LTD, Taipei, Taiwan) at a 0.01 g/ml concentration. Our preliminary experiments revealed that the thickening agent had no discernible effect on blood glucose levels in mice.

Each B6 male mouse was uniquely identified via ear notching and randomly assigned to either the experimental group (receiving dragon blood) or the control group (receiving only the thickening agent). Each group consisted of eight animals. At seven weeks of age (week 0), the mice underwent handling training to acclimate to restraint and blood was collection procedures from the lateral saphenous vein. Basal blood glucose measurements were obtained for all mice.

By the 8th week of age (week 1), animals were orally administered 600 µl of either the thickening agent or an equivalent 31 mg freshly prepared dragon blood suspended in 600 µl of the thickening agent solution. Oral administration was performed daily between 1:30 and 2:30 pm using an 18-gauge ball-tipped gavage needle and continued for a total of 10 weeks.

The blood glucose levels were monitored weekly. To ensure accurate measurements, rodent chow and corn cub-based bedding were removed six hours before assessment. Blood samples were collected from the lateral saphenous vein by puncturing it with a lancet at a 90-degree angle. Glucose levels were measured using the GlucoSure STAR High-Performance Blood Glucose Monitoring System (AB-201G; ApexBio, Hsinchu, Taiwan). After collecting a sufficient volume (> 2 ul) of blood, gentle pressure was applied using clean gauze to the puncture site until bleeding ceased. Body weight was measured weekly throughout the 10-week experiment.

### HPLC analysis

A dragon blood solution (83 mg/ml) containing a thickening agent at a concentration of 0.01 g/ml was utilized for animal feeding. The solutions were collected both before and after being stored for one week at 4 °C. Subsequently, they were diluted to a concentration of 10 mg/ml using methanol and clarified through centrifugation at 4000 ×g for 5 min. Before injection, the samples underwent filtration using a 0.22 μm syringe filter. HPLC analysis was conducted to examine the reference standard compound, dracorhodin, present in dragon blood from *D. draco*, employing a Hitachi L-7000 HPLC system. The analytical conditions were reported previously (Ho et al. 2016). Analysis was performed using a NUCLEODUR 100-5 C18 ec, 5 μm, 250 × 4.6 mm analytical column (MACHEREY-NAGEL GmbH & Co. KG, Dueren, Germany).

### Measurement of UV-visible spectra of dragon blood solution

As described above, samples (10 µl) intended for HPLC analysis were mixed with 490 µl methanol. Following this, spectra were then recorded using a BioMate 3 S spectrophotometer (Thermo Fisher Scientific Inc., Waltham, MA, USA), with methanol serving as the blank.

## Results

### In vitro glucose uptake assay

**Fig. 2:**
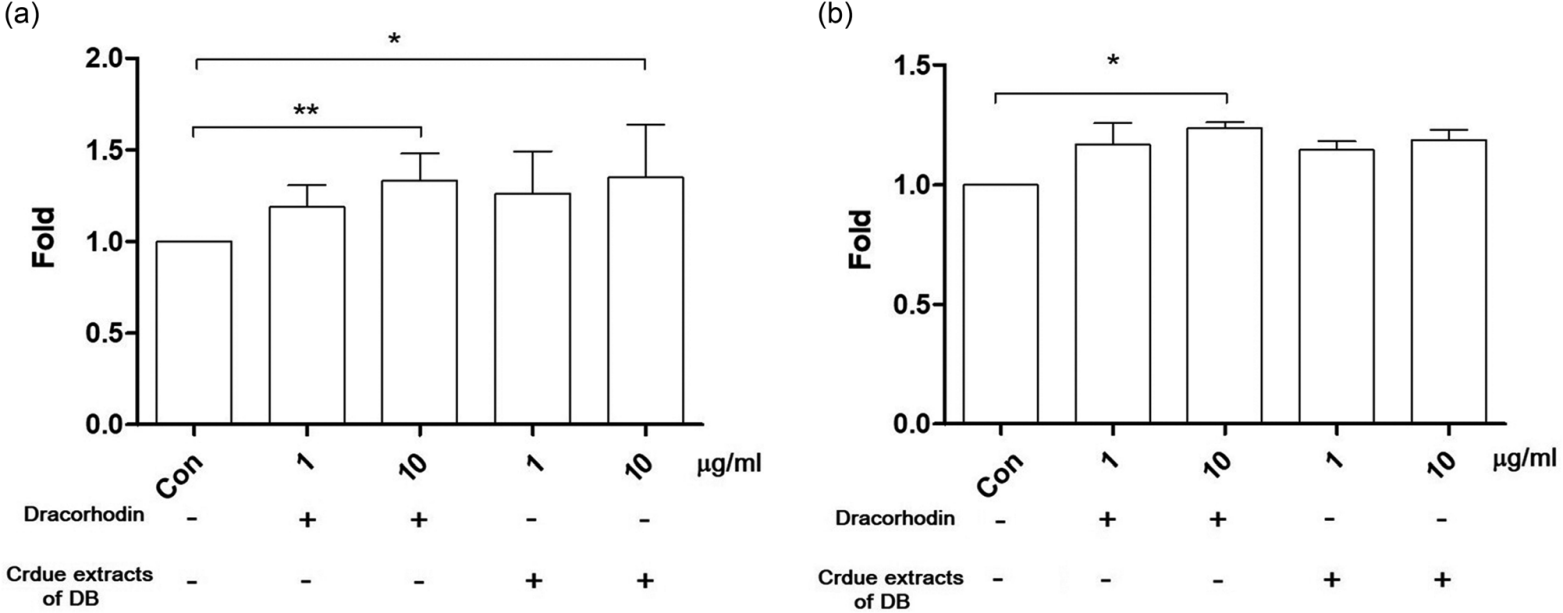
Dracorhodin and crude extracts of dragon blood promote glucose uptake in (**A**) C2C12 cells and (**B**) RASMC using 2-NBDG assay

Considering the pivotal role of glucose uptake by insulin-sensitive tissues, including skeletal muscle, liver, and adipose tissue in glucose homeostasis regulation (Balasubramanian et al. 2014), our investigation focused on evaluating the impact of dracorhodin and crude extracts of dragon blood on C2C12 cells (a perpetual muscle cell line) and RASMC (a primary culture of smooth muscle cells). Cells were subjected to a 24 h incubation period with either 1 or 10 µg/ml dracorhodin or crude extracts of dragon blood. Our observations revealed a significant increase in fluorescence intensity in both dracorhodin- and dragon blood crude extract-treated cells compared with the control group. Dracorhodin (1 µg/ml) increased glucose uptake by 133.3% ± 14.8% in C2C12 cells and 123.5% ± 4.4% in RASMC, and crude extracts of dragon blood (10 µg/ml) by 135.1% ± 28.8% in C2C12 cells and 118.6% ± 7.5% in RASMC. This substantial enhancement strongly suggests that both dracorhodin and crude dragon blood extracts effectively stimulated glucose uptake in RASMC and C2C12 cells (Fig. 2A and B).

Notably, both 10 µg/ml dracorhodin and crude extracts of dragon blood exhibited a similar effect on glucose uptake in both cells. However, the dracorhodin content in the dragon blood samples that we used is 1.3%. In other words, the concentration of dracorhodin in 10 µg/ml crude extracts of dragon blood sample is 0.13 µg/ml, which is significantly lower than 10 µg/ml pure dracorhodin. This result strongly implies the presence of other unidentified components in the crude dragon blood extracts that also possess glucose uptake-stimulating properties.

### Effects of AMP-activated protein kinase (AMPK) and phosphoinositide 3-kinase (PI3K) pathway inhibitors on glucose uptake in C2C12 myotubes

**Fig. 3:**
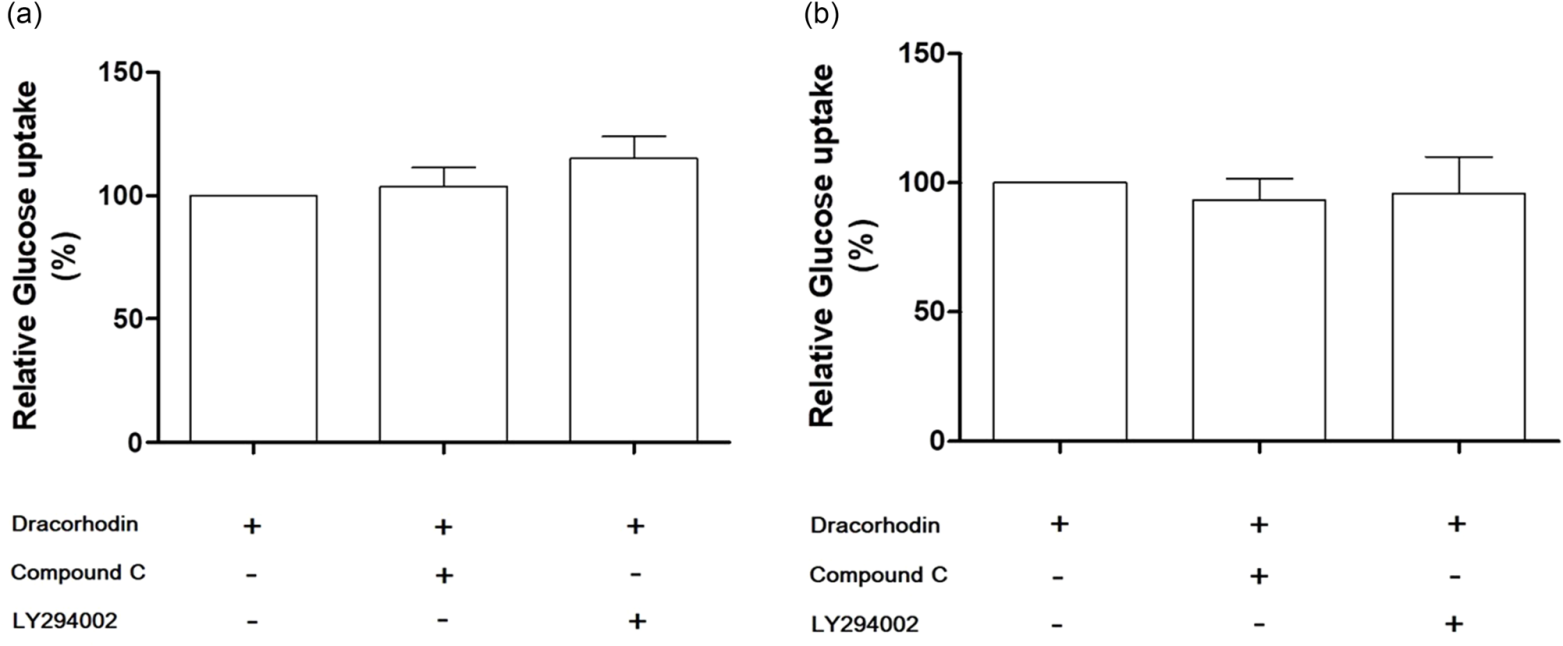
Effects of inhibitors on glucose uptake induced by (**A**) dracorhodin and (**B**) crude extracts of dragon blood in C2C12 myotubes. For the glucose uptake assay using several inhibitors, after pre-incubation in DMEM with a low-glucose concentration in the presence or absence of 40 µM LY294002 or 4 µM compound C

To elucidate the molecular mechanism underlying the effect of dracorhodin and crude extracts of dragon blood on glucose uptake, we pretreated C2C12 myotubes with compound C and LY294002, AMPK and PI3K inhibitors, respectively. Neither of these inhibitors attenuated the 2-NBDG absorption induced by dracorhodin or the crude extracts of dragon blood (Fig. 3A and B). Notably, 40 µM LY294002 slightly enhanced the 2-NBDG dracorhodin-induced absorption. Consequently, the molecular mechanism responsible for stimulating glucose uptake by dracorhodin or crude dragon blood extract remains unclear.

### In vivo animal studies

**Fig. 4:**
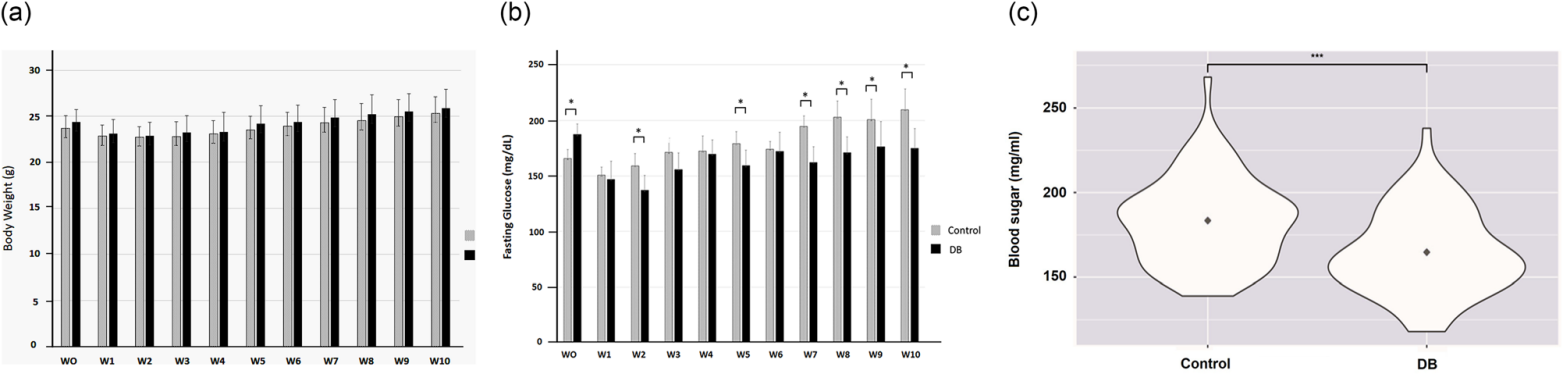
Effect of dragon blood crude extracts on the fasting blood glucose level in C57BL/6JNarl male mice model, (**A**) body weight, (**B**) fasting blood glucose levels, and (**C**) violin plot. (**A**) The weekly body weight of the C57BL/6JNarl male mice orally administered dragon blood crude extract suspension (DB) and control solution for ten weeks. The body weight of male B6 mice showed a steady increase during the experimental period, with no significant weight differences observed between the experimental (*N* = 8) and control groups (*N* = 8) from Week 0 to Week 10. (**B**) Fasting blood glucose of the C57BL/6JNarl male mice orally administered with dragon blood crude extract suspension solution compared to the control solution over ten weeks. At seven weeks of age (Week 0), animals were randomly assigned to either the dragon blood crude extract-treated (DB) or the control group and were trained to be accustomed to handling and restraining. The control and the experimental groups showed significant differences at weeks 0, 2, 5, 7, 8, 9, and 10. (**C**) The violin plot of fasting blood glucose is significantly lower in the BD-treated group than in the control group. The fasting glucose from the DB and the control group from W1 to W5 and form W7 to W10 (W6 data removed). The data were expressed as mean SD (*N* = 8). Dragon blood crude extract-treated group (Mean ± SD = 165.35 ± 24.60), and control group (Mean ± SD = 182.78 ± 24.81) with *p*-value = 1.541 × 10^− 5^

The body weight of male B6 mice, showed a steady increase during the experimental period, with no significant weight differences observed between the experimental (*N* = 8) and control (*N* = 8) groups from week 0 to Week 10 (Fig. 4A).

Before initiating oral administration of the dragon blood crude extract suspension solution (week 0, at 7th weeks of age), the mice underwent training to acclimate to restraint and lateral saphenous vein blood collection. The training period is important to prevent anxiety-induced blood glucose elevation. The fasting blood glucose levels were measured weekly. Surprisingly, even though the animals were randomly assigned to the experimental or control groups, the average blood glucose levels were significantly higher in the experimental group at week 0 (Fig. 4B), although no treatment was administered to either group.

Compared with the control group, the experimental group showed a decreasing trend in fasting blood glucose levels from the second to the tenth week. At weeks 1, 3, and 4, no significant differences in fasting blood glucose levels were observed except at week 2. From weeks 5 to 10, most weeks showed significant differences, except for week 6 (Fig. 4B). On week 6, owing to manpower issues, the oral administration task was temporarily handed over to a different staff set, which led to miscommunication. On week 6, all dragon blood crude extract suspension solutions needed for that week were prepared at the beginning of the week. Consequently, the blood glucose-lowering effects of the dragon blood crude extract were diminished at week 6, likely due to oxidation of the natural products.

We further performed a *t-test* analysis by combining blood glucose measurements regardless of the week and comparing the experimental and control groups. We excluded measurements from week 6 because of dragon blood preparation issues during that week. The blood glucose level of the group treated with dragon blood was significantly lower than that of the control group (*p* < 0.05, Fig. 4C).

**Fig. 5:**
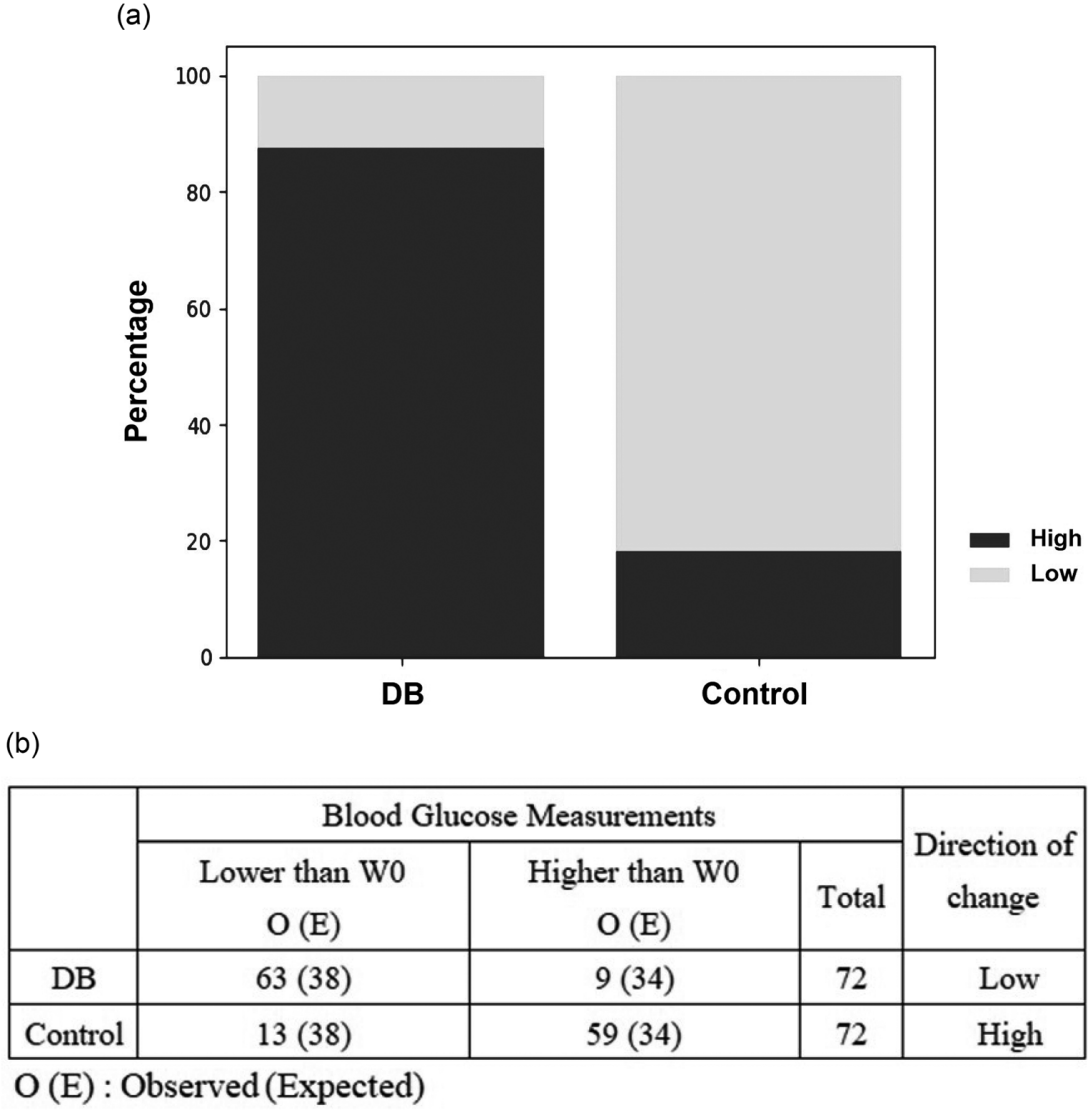
Pearson’s Chi-squared test of the blood glucose effect of the dragon blood-treated and control groups. Each week’s blood glucose measurement for a given individual between the experimental and control groups was subtracted from the measurement taken from W0. (**A**) Each blood glucose measurement was classified into the “low” group if < W0; or into the “high” group if > W0. (**B**) The Chi-square test results indicated that the dragon blood-treated group is statistically significant (*p*-value = 2.855e-16 < 0.05), indicating the blood glucose lowering effect was non-random even. The odds ratio of lower blood glucose in the BD group compared to the control group was 31.77

Pearson’s chi-square test with Yates continuity correction was also performed (Fig. 5). Each week’s blood glucose measurement for a given individual in the experimental and control groups was subtracted from the measurement obtained at week 0. Each measurement was classified into the “low” group if the glucose level was < W0; or into the “high” group if it was > W0. The odds ratio for lower blood glucose levels in the BD group compared to the control group was 31.77.

### HPLC analysis and UV-visible spectra of dragon blood solutions

**Fig. 6:**
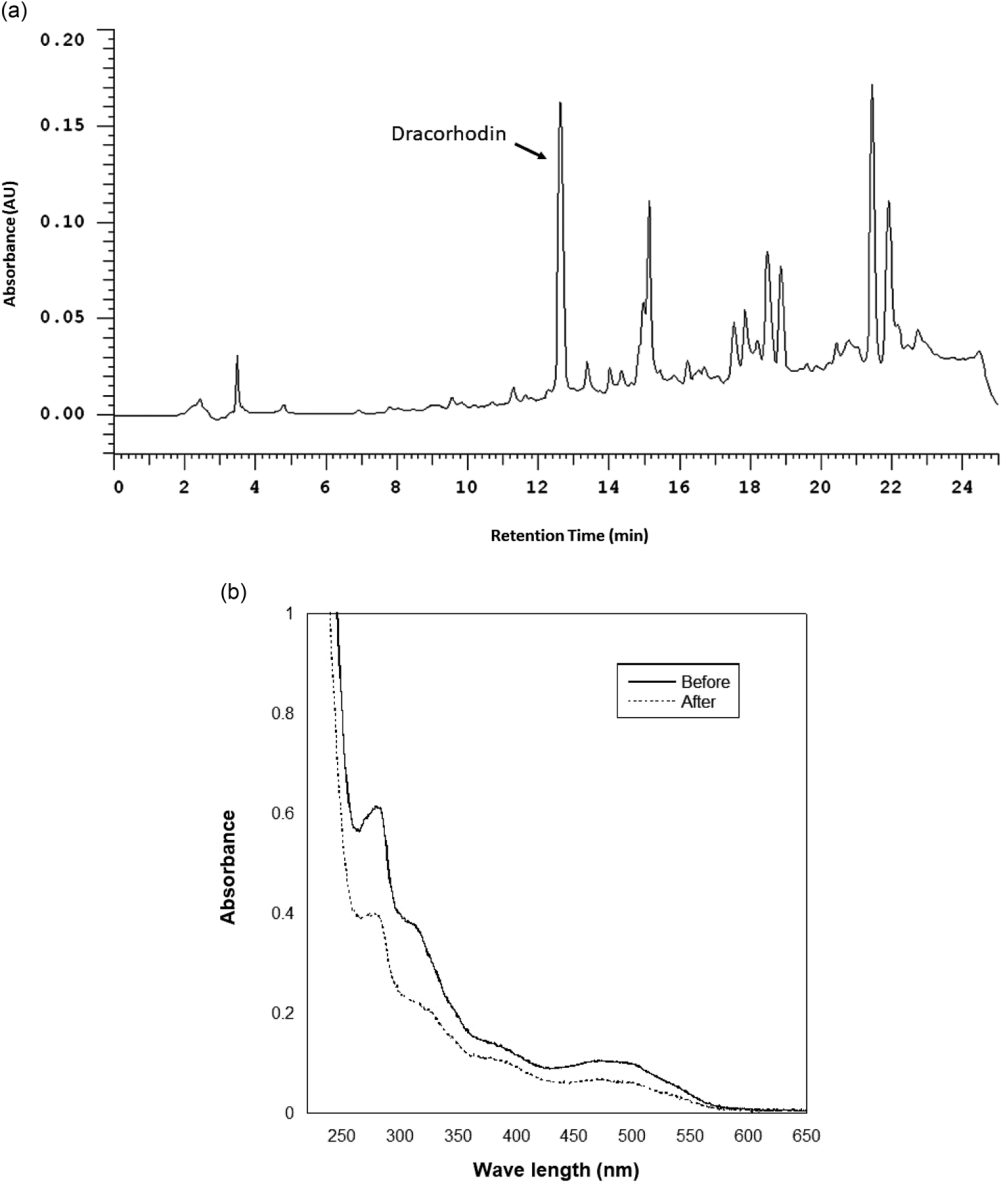
Analysis for animal-fed dragon blood solutions. (**A**) HPLC analysis, depicting the peak of the reference standard of dragon blood, dracorhodin; (**B**) UV-Visible spectra of dragon blood solution, before and after storage for one week at 4 °C

To assess whether storage at 4 °C for one week might cause the dragon blood solution to be oxidized and changed, both HPLC analysis and measurement of the UV-visible spectra of the dragon blood solution were conducted. As shown in Fig. 6A, the reference standard compound, dracorhodin, eluted around 12.6 min, with its peak area reduced to only 82.4% left after storage. This outcome suggests that dracorhodin might not be the primary molecule responsible for lowering blood sugar in vivo. In addition, UV-visible spectra of dragon blood solution, before and after storage, were recorded in the wavelength range of 220–650 nm (Fig. 6B). Evidently, the solution’s absorbance decreased after one week of storage. Furthermore, the absorbance ratio at various wavelengths between before and after storage exhibited alterations. For instance, the absorbance at 240 nm, 280 nm, 400 nm, and 540 nm was only 72.9%, 64.1%, 79.9%, and 55.2% left, respectively, after storage. All results indicate that oxidation indeed occurs after one week of storage at 4 °C.

## Discussion

In the 2015 edition of the Pharmacopoeia of the People’s Republic of China, a comprehensive catalog comprising 1,491 prescription formulations in the traditional Chinese medicine section was documented. Among these formulations, 20 included dragon blood, with seven of these exclusively for designated for oral administration and eight designated for topical use only. In addition, five formulations were approved for both external and oral use. Most formulations are intended to promote blood circulation, alleviate blood stasis, treat bruise injuries, reduce swelling and pain, address putrefaction, and promote muscle growth.

Our results clearly demonstrated that both dracorhodin and crude extracts of dragon blood from *Daemonorops draco* can stimulate glucose uptake in both the murine muscle cell line C2C12 and primary rat aortic smooth muscle cells. Dracorhodin is not the only active compound in the crude extract of dragon blood from *D. draco*. Among the 20 dragon blood-containing formulations listed in the Pharmacopoeia of the People’s Republic of China, 12 of them were orally administered. This stimulatory effect on glucose uptake was also observed in B6 mice, which can account for the efficacy of dragon blood, as described above. Accordingly, previous studies have consistently shown that dragon blood from *D. draco* can attenuate high glucose-stimulated oxidative stress and endothelial dysfunction in human umbilical vein endothelial cells (Chang et al. 2014). Consequently, these findings may shape future treatment approaches and shed light on the development of new therapies for cardiovascular conditions and metabolic syndromes.

## Data Availability

Data or material will be made available on request.
